# Single Tablet Regimen Usage and Efficacy in the Treatment of HIV Infection in Australia

**DOI:** 10.1155/2015/570316

**Published:** 2015-10-13

**Authors:** B. Armstrong, D. J. Chan, M. J. Stewart, D. Fagan, D. Smith

**Affiliations:** ^1^Graduate School of Medicine, University of Wollongong, Wollongong, NSW 2522, Australia; ^2^St George Hospital, South East Sydney Local Health District, Sydney, NSW 2217, Australia; ^3^Albion Centre, South East Sydney Local Health District, Sydney, NSW 2010, Australia; ^4^School of Public Health and Community Medicine, University of New South Wales, Sydney, NSW 2052, Australia; ^5^Discipline of Pharmacy, Faculty of Health, University of Canberra, Canberra, ACT 2617, Australia

## Abstract

Single tablet regimens (STRs) for HIV infection improve patient satisfaction, quality of life, medication adherence, and virological suppression compared to multitablet regimens (MTRs). This is the first study assessing STR uptake and durability in Australia. This retrospective audit of all patients receiving an STR (*n* = 299) at a large Sydney HIV clinic (January 2012–December 2013) assessed patient demographics, treatment prior to STR, HIV RNA load and CD4 during MTR and STR dosing, and reasons for STR switch. 206 patients switched from previous antiretroviral treatment to an STR, of which 88% switched from an MTR. Reasons for switching included desire to simplify treatment (57%), reduced side effects or toxicity (18%), and cost-saving for the patient. There was no switching for virological failure. Compared to when on an MTR, patients switching to an STR had significantly lower HIV RNA counts (*p* < 0.001) and significantly higher CD4 counts (*p* < 0.001). The discontinuation rate from STR was very low and all patients who switched to an STR maintained virological suppression throughout the study duration, although the study is limited by the absence of a control group.

## 1. Introduction

Advances in the development of antiretroviral agents (ARVs) for the treatment of HIV have reduced morbidity and mortality from AIDS [1]. Over the past 2 decades, more potent, less toxic ARVs have been developed and treatment regimens of some 20 pills per day are now reduced to once-daily, single tablet regimens (STRs) incorporating 3 ARVs. STRs may improve adherence by reducing pill burden and thus prevent the development of drug-resistance mutations to individual ARVs in multitablet regimens (MTRs) [2].

There is also an association between ARV pill burden and risk of hospitalisation. Cohen et al. [3] note that patients receiving an STR had significantly better rates of adherence compared to those receiving MTRs, and this translated to lower rates of hospitalisation. Overall costs were higher with patients receiving MTRs due to higher pharmacy and inpatient related care costs [3]. A recent Italian study concluded that the STR not only resulted in better adherence, but added €4541.00 lower cost-effectiveness ratio per QALY in comparison with the MTR with a 17% lower cost in favour of the STR [4].

STRs are generally associated with better adherence and higher viral suppression [3, 5, 6] and patient-reported outcomes (i.e., better tolerability and satisfaction with the STR) are correlated with better quality of life [5, 7]. That said, adherence to ARVs is not necessarily improved by STRs where there are differences in access to care, for example, rural versus urban-based patient populations [8]. STRs also improve adherence in patients who have other chronic diseases requiring multiple treatments [9, 10]. Indeed, the World Health Organization (WHO) recommends STRs for the treatment of hypertension, tuberculosis, and HIV infection [11].

There are 4 STRs approved for use in Australia, that is (in order of approval), (1) tenofovir/emtricitabine/efavirenz (*Atripla*); (2) tenofovir/emtricitabine/rilpivirine (*Eviplera/Complera*); (3) tenofovir/emtricitabine/elvitegravir/cobicistat (*Stribild*); and (4) dolutegraivir/abacavir/lamivudine (*Triumeq*). The STRs “recommended” for initial therapy in current Australian HIV treatment guidelines include* Atripla* and the integrase inhibitor based* Stribild* and* Triumeq*, with* Eviplera/Complera* listed as “alternative” regimen in patients with HIV RNA < 100,000 copies/mL and patients whose CD4 count exceeds 200 cells/*μ*L [12].

To our knowledge, no Australian study hitherto has compared the efficacy of STRs with MTRs, nor assessed the reasons patients switch from STRs to MTRs.* Atripla* and* Eviplera/Complera* were the only 2 STRs available when this study was conducted.* Atripla* is effective at achieving and maintaining virological suppression compared with MTRs [13]. In the STAR study,* Eviplera/Complera* was statistically noninferior to* Atripla* in relation to virological efficacy when baseline HIV RNA ≥100,000 copies/mL and statistically superior when baseline HIV RNA <100,000 copies/mL [14].

## 2. Materials and Methods

Between 1 January 2012 and 31 December 2013, we conducted a retrospective audit of the medical and ARV dispensing records for all patients receiving an STR prescribed and managed by a clinic doctor during the said period (patients who were dispensed medication prescribed by external doctors were excluded from the analysis). The study was conducted at Albion Centre, a WHO Collaborating Centre for Capacity Building and Health Care Worker Training in HIV/AIDS Care, Treatment and Support, and Australia's largest public, multidisciplinary specialist HIV treatment centre, located in metropolitan Sydney.

Patients completed a brief questionnaire concerning demographics, current STR, and previous ARV regimen (if not treatment-naïve). They were asked to specify the* main* reason for switching to an STR according to the following predefined reasons: desire for an STR (convenience), once-daily dosing, and improved toxicity, previously on a clinical trial (trial ended), or to state their own reason.

We established the number of Albion Centre patients receiving STRs including the proportion and reasons commenced on an STR as treatment-naïve patients, switched from an MTR to an STR, switched from an STR to another STR, and switched from an STR to MTR. We compared the HIV RNA and CD4 count of those on STRs to those on MTRs to deduce differences in relative virological efficacy and immune response.

Patients either* initiated* an STR as a treatment-naïve patient or* switched* from previous treatment to an STR prior to the study period; that is, all patients were receiving STRs during the study period and STR switches to an MTR “discontinuations” were then evaluated to calculate STR survival (STR switch to an alternative STR was not classified as a treatment discontinuation for statistical purposes). Mean HIV RNA and CD4 count were obtained for the duration of previous therapy with MTR and STR from the medical record.

Data were analysed using SPSS version 22.0. Regarding statistical analyses, difference in mean HIV RNA during treatment with MTR versus STR was calculated using* Wilcoxon's Sign-Rank test*; difference in mean CD4 count during treatment with MTR versus STR was calculated using a two-tailed paired *t-test*; difference in observed proportion of patient with HIV RNA >20 copies/mL was calculated by* McNemar's test*; and mean estimated STR survival during the 24-month study period was calculated by means of a* Kaplan-Meier* survival plot.

The postswitch HIV RNA and CD4 count were taken as the last available results in the medical record preceding 31 December 2013. No patients were switched for reason of virological failure. Although the time from the initiation of STR until the end of study end-date varied for each patient, this was deemed the best indication of the efficacy of the STR for the purpose of our analysis. Ethics approval was obtained from the South Eastern Sydney Local Health District Human Research Ethics Committee.

## 3. Results

A total of 299 patients were receiving an STR during the study period. This represented approximately 25% of all ARV prescriptions dispensed through our pharmacy at the time. 96% of patients were male and 4% were female. The cohort mean age was 42 years (range 19–73); 10% (29 patients) were hepatitis C antibody positive, 6% (18 patients) reported previous injecting drug use, and 5% (14 patients) were hepatitis B core antibody positive.

Two-thirds of patients (193) had previously received ARVs prior to enrolment and one-third (106 patients) were treatment-naïve. HIV RNA and CD4 counts obtained on the day of first prescription of an STR, if available, or immediately preceding the STR initiation, were used to define the “HIV RNA at switch” and “CD4 at switch” for the purpose of statistical analyses.

During the study period, 206 patients* switched* from their previous ARV regimen to an STR (14 previously treatment-naïve, 192 previously treated). The details of previous ARV regimen and switch to an STR are shown in Table 1. Approximately half the patients switched their previous treatment to* Atripla* and the other half to* Eviplera/Complera*. The majority (88%, 182 patients) had switched from an MTR, 11% (23 patients) switched from an STR, and 1 patient, who had received an MTR but stopped treatment prior to the study period, was commenced on an STR (this patient was counted as a “switch” for the purpose of the analysis).

### 3.1. MTR Switch to STR

One hundred and eighty-three patients received an MTR as previous treatment for a mean duration of 6.25 years before switching to an STR; 56% (103 patients) requested the switch in order to* simplify their ARV treatment* (i.e., convenience of a single tablet, once-daily dosing, cost reduction); 17% (32 patients) switched due to* side effects or toxicity* (e.g., hypercholesterolaemia, gastrointestinal symptoms); and 5% (10 patients) switched for some other reason (namely, immigration to Australia from countries with limited ARV access, initiation of hepatitis C treatment, reinitiation of ARV after prolonged interruption, and previous enrolment in a clinical trial). There was no reason documented for the MTR switch for 21% of patients.

Seventy-four percent (136 patients) had HIV RNA <20 copies/mL at the commencement of the STR. Of the 95 patients who switched to* Atripla*, 70% (67 patients) were previously taking tenofovir/emtricitabine (*Truvada*) and efavirenz as 2 separate ARV tablets. Of the 82 patients who switched to* Eviplera/Complera*, 73% (60 patients) were previously receiving a* Truvada* backbone with either nevirapine (14/60 patients), or atazanavir plus ritonavir (21/60 patients), or lopinavir/ritonavir (*Kaletra*, 8/60 patients), or raltegravir (10/60 patients). Remaining patients received a variety of ARVs.

### 3.2. STR Switch to STR

Twenty-three patients receiving* Atripla* switched to* Eviplera/Complera* during the study period. 56% (13 patients) switched due to typical efavirenz toxicity (neurocognitive symptoms, rash, or lipid elevation) and 13% (3 patients) switched due to the convenience of taking their medication with meals. There was no reason for switching STRs documented for 8 patients. No patients switched from* Eviplera/Complera* to* Atripla*. Notably, 10% (30/299 patients) of all switches were from* Atripla* due to toxicity. This includes the 23 patients switching to* Eviplera/Complera* and 7 other patients who switched from* Atripla* to an MTR (discussed below).

### 3.3. STR Survival

The mean pretreatment HIV RNA as well as CD4 count for treatment-naïve patients was 122,414 copies/mL and 414 cells/*μ*L, respectively. Mean HIV RNA and CD4 count were calculated for duration of therapy with an MTR (i.e., before switch) and an STR (i.e., time from* initiation* for treatment-naïve patients or* switch* for treatment-experienced patients). Mean HIV RNA and CD4 differed during treatment with MTRs and STRs. Mean HIV RNA during treatment with MTR was significantly higher compared to that during treatment with an STR, being 5454 and 1103 RNA copies/mL, respectively (*Z* = −4.718; *p* < 0.001,* Wilcoxon's Sign-Rank test*). (Treatment-naïve patients were not included in the pre- and postswitch analysis. Only those switching were analysed to obtain the aforementioned *p* values.)

Mean CD4 count during treatment with MTR was significantly lower than that with an STR, mean CD4 being 554 cells/*μ*L and 620 cells/*μ*L, respectively (*p* < 0.001, 2-tailed paired* t-test*). At the time ofswitch from an MTR to an STR, or an STR to an STR, the proportion of patients with HIV RNA >20 copies/mL was significantly higher with MTRs compared to STRs [69% (38 patients) versus STRs 58% (14 patients), resp. (*p* < 0.001,* McNemar's test*)].

**Figure 1:**
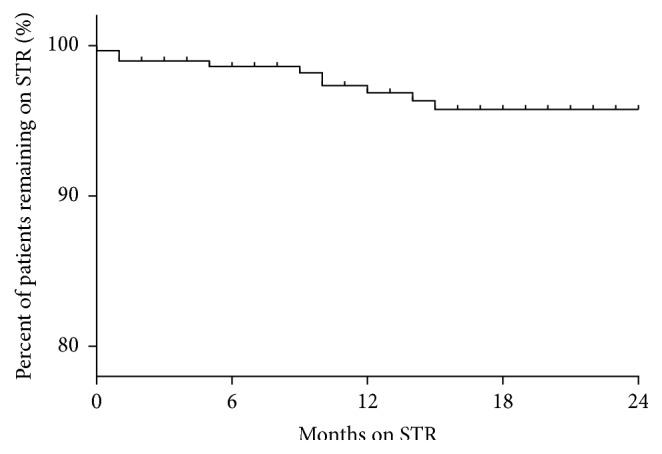
Durability of STRs over 24 months (*n* = 299).

The benefits of convenience and improved adherence with STRs are meaningless if STRs cannot maintain virological suppression (durability) which is critical in preventing HIV disease progression and development of viral resistance. In terms of survival on an STR, all patients remaining on an STR maintained virological suppression throughout the duration of the study (HIV RNA < 20 copies/mL). The mean estimated survival time on an STR was 23.3 months calculated by* Kaplan-Meier* analysis (*standard error* = 0.204; 95% CI 22.95–23.75), whilst the STR discontinuation rate was low overall at 3% (10/299) (Figure 1).

Of the 10 patients who switched off an STR to an MTR during the study period, there were 7* Atripla* switches due to efavirenz toxicity (neurocognitive symptoms, rash, lipid elevation, and abnormal liver function from hepatitis C coinfection) and 3* Eviplera/Complera* switches due to declining* e*GFR, insomnia, rash, or abdominal pain. For those that switched from an STR to MTR, choices for regimens included a* Truvada* backbone plus either raltegravir or darunavir/ritonavir; or an abacavir/lamivudine (*Kivexa/Epzicom*); or zidovudine/lamivudine (*Combivir*) backbone plus rilpivirine.

## 4. Discussion

*Atripla* and* Eviplera/Complera* were the only STRs recommended by ARV guidelines and licensed in Australia at the time this study was conducted. To our knowledge, this is the first study of STR uptake and durability conducted in Australia. Most patients (97%) remained on their STR over the 2-year observation period. Ten patients (3%) ceased their STRs, due to toxicity or tolerability issues: renal decline secondary to the tenofovir component of the STRs [15], abnormal blood lipid profiles, and for several patients complaining of drug-induced rash or insomnia, all known side effects of efavirenz [16–18]. Efavirenz was ceased in 3 cases due to viral hepatitis with subsequent hepatic impairment, which has been shown to affect plasma levels of efavirenz [19]. Since this study was undertaken, the integrase inhibitor based STRs* Stribild* and* Triumeq* have come to market and may have been an appropriate alternative STR for the 10 patients in the study who switched from* Atripla* or* Eviplera/Complera* to an MTR.

STR discontinuation rates vary across studies. Fabbiani et al. [20] showed a discontinuation rate of ARV therapy that included efavirenz of 40.6% for MTR versus 17.1% on STR over 4 years, whereas, in their study of patients taking* Eviplera/Complera*, Pinnetti et al. [21] showed a discontinuation rate of 13.2% at 12 months. In a cohort analysis, Rappold et al. [22] described a discontinuation rate <5% over 2 years, which approximates the 1% discontinuation rate for the patients in our study over the same duration. The reasons for the low discontinuation rate in our study remain unclear but may relate to differing follow-up durations and designs of other studies, relative tolerability of evaluated regimens, the financial benefit of STRs in Australia, or our multidisciplinary model of patient care that focuses on maximising ARV adherence through patient empowerment and expeditious side effect management.

CD4 counts rise after initiation of ARVs in most patients who achieve an undetectable HIV RNA load [23]. A limitation of our study is that we cannot determine whether increases in CD4 in patients switching to STR are due to the STR* per se*, increased compliance, or merely natural immune recovery over time. Another limitation with our review, apart from its retrospective design, is that we were unable to compare the 2-year outcomes of patients initiating an STR with a similar cohort initiating an MTR; that is, there was no control group in the analysis. Nonetheless, improved virological suppression was clearly demonstrated, with significantly more patients having undetectable or decreased HIV RNA loads whilst receiving an STR in our cohort. Finally, our analysis pooled the treatment-naïve and MTR subjects which may have added bias to the results in terms of relative survival.

The often-chaotic lifestyles associated with recreational intravenous drug use were initially postulated to be a predictor of STR usage; however only a relatively low level of injecting drug use was reported in our cohort. It must be noted, however, that our records rely on self-reporting by the patient in questionnaire form on presentation to the clinic. It is unlikely that the numbers recorded represent a reliable estimate of intravenous drug use in this community.

## 5. Conclusion

STRs are an integral option in the treatment of HIV; however, the initiation of a particular STR, or decision to switch to one, may depend on factors such as persistence of early ARV combinations (and patient preference* not* to change), improved awareness of cumulative toxicities associated with specific ARVs, the relative strength of the genetic barrier to resistance in poorly adherent patients, and other factors, such as socioeconomic disadvantage, comorbidities, and interactions with prescribed and illicit drugs.

In this study,* Atripla* and* Eviplera/Complera* were associated with improved CD4 counts and lower HIV RNA loads compared to MTRs. Once stabilised on an STR, there were few discontinuations and virological suppression was sustained. Most switches from an MTR to an STR were due to patient factors, such as pill burden, convenience, tolerability, and/or cost, whilst there were very few switches from one STR to another due to predicted side effects or dietary limitations. Our study suggests that once patients are initiated on an STR they are likely to remain on it over a medium term whilst maintaining virological suppression. As such, STRs are an effective option for many patients. The relative benefits of the newer integrase inhibitor based STRs may further extend these advantages, but this remains to be tested.

## Figures and Tables

**Table 1:** Disposition of ARV switch to STR.

	*N*
Patients receiving STR	299
Patients switched to STR	206
Previous regimen type	
MTR	182
STR	23
Treatment interruption	1
STR switched to (from previous regimen)	
*Eviplera/Complera*	111
From MTR	83
From *Atripla*	23
From medication trials^*∗*^	4
From prolonged treatment interruption	1
*Atripla*	95
From MTR	93
From medication trials^*∗*^	2

^*∗*^Patients switched from medication trials categorised as MTR for statistical analysis.
